# Integrating rare diseases into Africa’s digital health strategies

**DOI:** 10.1371/journal.pdig.0001073

**Published:** 2025-11-06

**Authors:** Silas Frank Gamba, Martha Magili

**Affiliations:** 1 Department of Public Health, University of Liverpool, Liverpool, United Kingdom; 2 Tanzania Human Genetics Organization, Dar es Salaam, Tanzania; Beth Israel Deaconess Medical Center, UNITED STATES OF AMERICA

## Introduction

Driven by the urgent need to address a high burden of disease with limited resources, many African nations are increasingly turning to digital solutions to bridge gaps in access, improve diagnostic accuracy, and enhance the efficiency of healthcare systems. This adoption is characterized by a focus on prevalent and high-impact conditions, with telemedicine, mobile health (mHealth), and artificial intelligence (AI) being deployed to tackle infectious diseases like malaria, HIV/AIDS, and tuberculosis, as well as emerging noncommunicable diseases [1,2]. For instance, AI-powered diagnostic tools, such as those in Nigeria, have improved the accuracy of tuberculosis diagnosis [3]. Beyond TB, AI algorithms are being used for diabetic retinopathy screening in countries like Kenya, enabling earlier detection and intervention for a leading cause of blindness. Similarly, low-cost smartphone-based telemedicine systems and AI image analysis are being applied to cervical cancer screening, showcasing digital health’s potential to address critical public health challenges [4].

While African digital health has prioritized high-burden diseases like infectious and other noncommunicable diseases, rare diseases which affect approximately 50 million people in Africa remain largely unconsidered within the landscape of Africa’s digital health innovations and policy strategies [5,6]. This lack of dedicated focus has created a landscape where rare disease patients remain invisible, their needs unmet by the very solutions intended to improve healthcare. It also reflects a fundamental disconnect between the potential of digital health to address complex medical challenges and the existing priorities of policymakers—ultimately hindering equitable healthcare development across the continent [7].

## The role of digital tools in rare disease management

The integration of digital tools into healthcare systems presents a transformative opportunity to address the multifaceted challenges associated with rare disease management, particularly within the African context. Research highlights some key areas where digital interventions can be transformative:

1. **Improving Diagnosis and Care Coordination:** Patients with rare diseases often encounter difficulties due to fragmented healthcare systems, including medical record systems. Digital health solutions, from centralized patient records and registries to mobile health (mHealth) apps, can streamline care coordination and empower patients to take a more active role in their own health management. Also, symptom-tracking apps and AI-based diagnostics can significantly facilitate earlier diagnosis, ultimately improving care efficiency and patient outcomes [8].
2. **Enhancing Information Access, Community, and Patient Support:** Social media platforms and online patient networks such as Canada’s RareConnect provide a space for patients to share experiences, receive emotional support, and access medical information. These networks are crucial for rare disease patients who often feel isolated due to the rarity of their conditions [9]. The scarcity of reliable medical information on rare diseases poses a significant challenge for patients and caregivers. Digital tools that compile expert-reviewed content and offer tailored patient education can empower individuals with rare diseases to better manage their conditions and make informed decisions. However, ensuring the credibility of these platforms and mitigating misinformation is essential [10].
3. **Enabling Real-Time, Interoperable Data for Surveillance and Care Improvement:** Digital platforms that enable interoperable data collection and integration—across electronic health records, registries, and patient-reported outcomes—are vital for enhancing rare disease surveillance, accelerating research, and improving care. Such infrastructure can also provide evidence to inform not only clinical decisions but also health planning and policy development [4,6].

## Gaps in digital health adoption for rare diseases in Africa

Many African nations have made efforts to address rare diseases and to advance digital health. However, some digital health strategies overlook rare diseases, while some rare diseases initiatives fail to incorporate the potential of digital health [11]. Tanzania, for example, in Tanzania’s national Call for Action on rare diseases, focuses on data collection, policy advocacy, public awareness, and research. However, there is no mention of a digital health strategy, which could enhance data collection, diagnosis, treatment, and patient support [12]. South Africa’s Digital Health Strategy 2019–2024 is another case in point. While it incorporates AI-based diagnostics and telemedicine, it fails to establish targeted policies addressing rare diseases, treating them instead as part of general healthcare objectives [11].

Another issue is the lack of structured patient registries, which are necessary to enhance patient monitoring and promote research and treatment innovations. Cameroon is a typical example of countries where lack of centralized data hampers strategic health planning, leading to neglect of people with rare diseases within the health systems [13]. A national rare disease registry would facilitate the systematic tracking of disease prevalence, earlier identification of people with RDs, and improved care and support services. It would also shape health policy and insurance frameworks.

Countries such as Canada and Brazil have successfully integrated rare disease registries into their national healthcare systems, ensuring patients receive specialized and multidisciplinary care. In Canada, most of the rare disease registries source data from already existing electronic health records (68%), clinician-reported data (68%), and medical chart abstraction (60%), providing data for real-world evidence and technology assessments [6,14]. Similarly, Brazil leveraged common digital tools, like REDCap for rare disease surveys, and implemented a standardized minimal data set, effectively identifying rare diseases and streamlining diagnosis and treatment processes. Also, the establishment of a national network with data collection was an important step in improving rare disease knowledge and closing care gaps in Brazil’s resource-limited settings. [15].

## Conclusion

Integrating rare diseases into Africa’s digital health strategies is essential for advancing healthcare equity across the continent. While economic constraints may hinder the independent development of national rare disease registries, these challenges can be addressed through phased implementation, integration with existing systems, and international partnerships. Drawing lessons from countries like Brazil, African nations can leverage open-source tools, regional data-sharing frameworks, and scalable governance models to develop secure, cost-effective solutions. By investing in AI diagnostics, telehealth, and patient-centered registries, and fostering collaboration among governments, healthcare providers, and advocacy groups, Africa can close the digital divide in rare disease care and ensure timely, equitable access to diagnosis and treatment.
